# The Effect of Diluted Penetration Enhancer in Nebulized Mist *versus* Liquid Drop Preparation Forms on Retrobulbar Blood Flow in Healthy Human Subjects

**DOI:** 10.3390/pharmaceutics4030377

**Published:** 2012-08-08

**Authors:** Sally Primus, Ingrida Januleviciene, Brent Siesky, Austin Gerber, Patrick Egan, Annahita Amireskandari, Lina Siaudvytyte, Ruta Barsauskaite, Alon Harris

**Affiliations:** 1 Eugene and Marilyn Glick Eye Institute, Indiana University School of Medicine, Indianapolis, IN 46202, USA; Email: sprimus@iupui.edu (S.P.); bsiesky@indiana.edu (B.S.); algerber@iupui.edu (A.G.); eganp07@gmail.com (P.E.); aamiresk@gmail.com (A.A.); 2 Department of Ophthalmology, Hospital of Lithuanian University of Health Sciences Kaunas Clinics, LT-50009 Kaunas, Lithuania; Email: ingrida.januleviciene@kaunoklinikos.lt (I.J.); lynciuke@gmail.com (L.S.); rutabars@gmail.com (R.B.)

**Keywords:** retrobulbar blood flow, color Doppler imaging, nebulized mist

## Abstract

The aim of this study was to compare the effects of nebulized mist and liquid drop applications on retrobulbar blood flow. A prospective, non-randomized clinical trial was used to collect data from 40 healthy human eyes. Color Doppler Imaging determined peak systolic (PSV) and end diastolic (EDV) blood flow velocities and resistance index (RI) in the ophthalmic artery after both applications. Measurements were taken at baseline and at 1 min post-treatment in both eyes with 5 min measurements in the treatment eye only. *p* values ≤ 0.05 were considered statistically significant. Mist application to treatment eye produced an increase in 1 min and 5 min PSV and EDV (0.001 < *p* < 0.03) and a decrease in 5 min RI (*p* = 0.01), with no significant changes in PSV, EDV or RI of control eye or in treatment eye 1 min RI (*p* > 0.05). Drop application to treatment eye produced an increase in PSV (*p* < 0.001) and EDV (*p* = 0.01) at 1 min, with an increase in control eye 1 min PSV and EDV (*p* = 0.03). There were no statistically significant changes in treatment eye PSV, EDV and RI after 5 min (*p* > 0.05). The use of nebulized mist may provide an effective alternative to liquid drop medication application.

## 1. Introduction

Disease treatment in the field of ophthalmology offers unique forms of drug delivery not seen in other fields of medicine, offering tailored treatment for ocular diseases that attempt to maximize local response while minimizing systemic distribution. The treatment of ocular disease relies heavily on the use of topically applied ophthalmic medications, with the liquid drip, or “drop”, formulation far out-weighing the other methods of therapy application. While success is achieved in many patients with drops, a significant proportion of patients have numerous difficulties with this method of drug 1delivery. Current alternatives to drop preparations include much more invasive methods, such as 1intravitreal injection and sub-Tenon’s infusion. However both of these methods carry risks such as 1endophthalmitis and bleeding and are not considered reasonable methods of drug delivery in many 1ocular diseases [1].

Topical administration through drops allows intraocular medication delivery through penetration of the cornea, sclera, and perhaps conjunctiva. However, intraocular medication delivery through drop form poses several problems. The anterior ocular surface has limited permeability, and medication is quickly diluted by the constant influx of tears and drainage to the nasal cavity through the lacrimal duct system [2,3]. In order to reach therapeutic levels, eye drops must contain much higher concentrations of active ingredients than what is required intraocularly. In an effort to combat the limited permeability, multiple chemical enhancers have been added to the solutions in order to irritate the ocular surface, enhancing penetration. Unfortunately, this irritation can become severe enough to cause significant damage and patient discomfort, often leading to discontinuation of the medication [4,5]. In addition, studies have shown that while chemical irritants increase drug permeability into the eye, they also increase the amount of medication delivered systemically, potentially leading to systemic side effects, as seen with beta-blockers [6]. In an attempt to prevent these complications, most current ocular pharmaceuticals have changed formulations to include less effective enhancers, resulting in decreased medication penetration.

Due to the significant impact of ocular irritation on achieving therapeutic goals, researchers have sought for a better understanding of the importance of chemical enhancers in ophthalmic medications. Previous research has investigated the physiological response of ocular irritants by evaluating changes in ocular blood flow. The goal of these studies was to show the effects of the irritants alone without the confounding effects of the active medications. These studies have shown that this physiologic response seen with ocular medications can be reliably be reproduced by using alkalescent (pH > 6.9) saponin liquid drops, giving a reliable way to observe the effects of irritants alone [7,8]. This method of using saponin liquid to observe the effects of irritation on ocular blood flow gives further insight into the complex nature of ophthalmic drug delivery. Many ophthalmic medications have shown to have effects on ocular hemodynamics irregardless of irritation, so using saponin liquid drops allows the study of the role of chemical irritants separate from the active medication used to treat disease.

However, ocular irritation is only one disadvantage of the drop preparations, which present with several other poor compliance factors. Drop application often requires positioning the bottle above the ocular surface, which physically difficult in a significant proportion of patients. This positional difficulty increases the risk of corneal abrasion from contact between the bottle and ocular surface, in addition to the possible over or under dosing of medication. It is because of these disadvantages of drop preparations that other methods of ocular medication delivery have been explored [9,10].

Development of a safe, simple and non-invasive application of ophthalmic medication would 1enhance patient compliance and overall success in treating ocular diseases. Application of ocular medications through a nebulized mist form has been investigated in an attempt to overcome the common difficulties seen with drop preparations.

Medication delivery through nebulized mist is a well-established method of disease treatment outside of ophthalmology, especially in treating respiratory diseases, such as asthma. Through the use of a nebulizing machine, a liquid form of a medication is transformed into a cloud of particles that can then be administered to the patient. Nebulized mist poses several advantages over drop preparation, including accurate dosing, economical use of active ingredients, decreased risk of corneal abrasion, and increased patient compliance due to less irritation and fewer physical difficulties in use.

The purpose of our study was to compare the effects of nebulized mist *versus* drop preparation of diluted penetration enhancer solution on retrobulbar blood flow in healthy human subjects.

## 2. Experimental Section

This study was a prospective, non-randomized clinical trial carried out at the Kaunas Clinics of the Hospital of Lithuanian University of Health Sciences. Twenty healthy human subjects comprised the study group. All study procedures conformed to the tenets of the Declaration of Helsinki, and IRB Committee Approval (number was BE-2-9) was issued by Kaunas Regional Biomedical Trials Ethics Committee. All patients signed informed consents prior to enrollment.

Patients underwent a standard screening exam to corroborate their healthy status, verifying the absence of systemic vascular and cardiovascular diseases, hypertension (BP > 140/90 mmHg, as measured by automated ambulatory blood pressure after 5 min rest according to good clinical practice guidelines) [11], diabetes, pulmonary diseases, cancer, or other life threatening conditions. Likewise, exclusion criteria consisted of any surgery within the past three months, any ocular pathology (including glaucoma, infectious or inflammatory ocular diseases), or any history of allergic conditions. Inclusion criteria included age greater than 18 years and visual acuity of 1.0 as measured by the Snellen Eye Chart.

Participation required understanding of the possibility for mild discomfort during the application of the diluted sterile penetration enhancer solution to the ocular surface [7,8].

This study was performed in two stages in one visit: the first entailed applying a diluted penetration enhancer solution in nebulized mist form, while the second phase used a diluted drop. The phases were separated by a one-hour rest period. Sterile penetration enhancer solution was made in 4:1 proportions of sterile distilled water and saponin liquid (consisted of water, sodium laureth sulfate, cocamidopropyl betaine, glycerin, ammonium xylenesulfonate, disodium phosphate, citric acid), respectively.

The first experimental stage consisted of applying the nebulized mist of the penetration enhancer solution to the right eye, while the left eye served as the control. During this first study part, the nebulizer was applied continuously to the open right eye for six 30-s intervals. Patients were allowed to blink between application intervals. One minute after the sixth 30-s interval was completed, the first set of measurements was taken, with the second set of measurements taken five minutes after application. This served to measure the almost immediate hemodynamic response as well as a delayed response. After one hour of rest, the diluted drop was applied to each patient’s left eye, with the right eye serving as a control. Following each application, patients were asked if there was any subjective discomfort.

Baseline measurements of ophthalmic artery (OA) blood flow velocities and resistance index were acquired for both eyes utilizing color Doppler imaging (CDI) (Accuvix V20 model, Medison), which was performed by one adequately trained professional to ensure consistency. During CDI examinations, subjects were examined in supine position, with the upper body tilted upward at about a 30-degree angle. The transducer and its 7.5 MHz linear probe were applied gently to the closed eyelid using a coupling gel, and care was taken to avoid applying any pressure to the eye. The peak systolic velocity (PSV) and end-diastolic velocity (EDV) of the OA were assessed in both control and treatment eyes at each time-point. Resistance index in the OA was then determined by calculating Pourcelot’s resistance index (RI) using the formula RI = [(PSV − EDV)/PSV]. This technique provides insight into the ophthalmic circulation [12,13,14,15] as it has been shown to yield reproducible [16,17,18] measurements of blood flow velocities.

OA blood flow parameters were measured again for both eyes 1 min after applying the nebulized mist and again 5 min later for the experimental eye only. For the liquid drop, measurements one minute after application (both eyes) and 5 min after application (treatment eye only) were recorded. Following each application, patients were asked if there was any subjective discomfort.

The statistical data analysis was performed using computer program SPSS 17.0 for Windows. All variables were defined by methods of descriptive statistics. The analysis of the quantitative variables included calculation of the mean and standard deviation (*x* ± SD). Means of continuous variables were compared by Student’s *t* test for independent samples. Mann–Whitney’s nonparametric test was used when the assumption of the data normality was rejected. Paired Samples *t* test was used to compute the difference between the two variables for each case to see if the average difference is significantly different from zero. The level of significance *p* < 0.05 was considered significant.

## 3. Results and Discussion

All 20 healthy human subjects completed all phases of the study. Gender distribution in the study was 6 females (30%), 14 males (70%). Mean age of the study population was 28 (1.6) years. Mean baseline systolic and diastolic blood pressures were 128 mmHg (SD 14 mmHg) and 73 mmHg (SD 8 mmHg), respectively; mean heart rate was 73 (SD 9) bpm. There were no statistically significant differences in baseline parameters when comparing treatment and control eyes.

A significant increase in PSV and EDV, as well as a decrease in RI, was found after application of diluted penetration enhancer mist in the treatment eye only when compared to baseline. Increases in PSV in treatment eye were found after 1 min (*p* = 0.003) and 5 min (*p* = 0.03) following mist application.

Similarly, treatment eye EDV increased after 1 min (*p* = 0.01) and 5 min (*p* < 0.001). The RI decrease was not significant at 1 min (*p* = 0.24) but reached significance at 5 min (*p* = 0.01). Control eye CDI parameters remained stable at 1 min. Differences in baseline and 1 min control eye measurements of PSV, EDV and RI were not significant (*p* > 0.05) (Table 1 and Table 2). Application of mist caused no discomfort.

**Table 1:** Stage 1: Baseline and mist comparison after 1 min and 5 min.

	Baseline	Experiment eye (Right eye) after mist	
		1 min	5 min
PSV (Mean ± SD)	30.4 ± 12.2	37.8 ± 9.9 ( *p* = 0.003) *	37.2 ± 7.9 ( *p* = 0.03) **
EDV (Mean ± SD)	6.0 ± 2.6	7.8 ± 3.2 ( *p* = 0.01) *	9.2 ± 3.9 ( *p* < 0.001) **
RI (Mean ± SD)	0.81 ± 0.05	0.79 ± 0.09 ( *p* = 0.24)	0.77 ± 0.07 ( *p* = 0.01) **

* Paired Samples *t* test. Significant level *p* < 0.05 (misting changes from baseline after 1 min); ** Paired Samples *t* test. Significant level *p* < 0.05 (misting changes from baseline after 5 min).

**Table 2:** Stage 1: Baseline comparison with the control eye after 1 min of mist.

	Baseline	Control eye (Left eye) after mist
		1 min
PSV (Mean ± SD)	31.4 ± 9.1	32.9 ± 6.7 ( *p* = 0.32)
EDV (Mean ± SD)	6.7 ± 2.6	6.8 ± 2.3 ( *p* = 0.86)
RI (Mean ± SD)	0.78 ± 0.07	0.79 ± 0.05 ( *p* = 0.64)

* Paired Samples *t* test. Significant level *p* < 0.05.

Application of dilute penetration enhancer solution through liquid drop preparation showed a statistically significant increase in PSV and EDV after 1 min in both control and experimental eyes when compared to baseline measurements. An increase in experimental eye PSV and EDV was seen just after 1 min (*p* < 0.01) and decreased to baseline level after 5 min (*p* > 0.05). The decrease in RI was not statistically significant after 1 min (*p* = 0.60) or after 5 min (*p* = 0.65) (Table 3 and Table 4). Control eye measurements revealed an increase in PSV (*p* = 0.03) and EDV (*p* = 0.03) at 1 min but no changes in RI from baseline (*p* = 0.27). Five-minutes measurements were not taken in control eyes. All patients receiving the drop reported discomfort with application.

**Table 3:** Stage 2: Baseline and drop comparison after 1 min and 5 min.

	Baseline	Experiment eye (Left eye) after drop	
		1 min	5 min
PSV (Mean ± SD)	31.4 ± 9.1	42.3 ± 10.6 ( *p* < 0.001) *	37.4 ± 12.1 ( *p* = 0.06)
EDV (Mean ± SD)	6.7 ± 2.6	9.5 ± 4.3 ( *p* = 0.01) *	7.9 ± 4.1 ( *p* = 0.22)
RI (Mean ± SD)	0.78 ± 0.07	0.77 ± 0.08 ( *p* = 0.60)	0.79 ± 0.08 ( *p* = 0.65)

* Paired Samples *t* test. Significant level *p* < 0.05 (dropping changes from baseline after 1 min); ** Paired Samples *t* test. Significant level *p* < 0.05 (dropping changes from baseline after 5 min).

**Table 4:** Stage 2: Baseline compared to controleye (right eye) after 1 min of drop.

	Baseline	Control eye (Right eye) after drop
		1 min
PSV (Mean ± SD)	30.4 ± 12.2	37.7 ± 9.6 ( *p* = 0.03) *
EDV (Mean ± SD)	6.0 ± 2.6	7.6 ± 3.4 ( *p* = 0.03) *
RI (Mean ± SD)	0.81 ± 0.05	0.80 ± 0.06 ( *p* = 0.27)

* Paired Samples *t* test. Significant level *p* < 0.05.

## 4. Conclusions

This hemodynamic response to ocular irritation has been demonstrated previously, showing an increase in retrobulbar flow ranging from 29.6% to 200% in response to saponin liquid drop application in healthy patients [5,6]. Ocular irritation is a known stimulus of the sphenopalatine ganglion complex, and this stimulation leads to increased cerebral blood flow. Parasympathetic post-ganglionic fibers arising from the sphenopalatine ganglion stimulate the lacrimal gland to produce tears, and it is suggested that this pathway is the efferent limb of the tear secretion reflex. Therefore, as ocular irritation occurs, the tear secretion reflex is elicited, which may induce an increase in ocular blood flow [19].

Our results demonstrated that the application of a diluted penetration enhancer solution increases retrobulbar flow via both traditional liquid drop and nebulized mist application methodologies. Of specific note, the nebulized mist application increased retrobulbar flow in the experimental eye only, while the liquid drop formulation increased retrobulbar flow in both the experimental and control eyes.

This difference is likely due to a more systemic response to the irritation caused by the liquid drop application, as patients reported significantly more discomfort to this preparation when compared to the nebulized mist. The irritation caused by the saponin liquid drop was expected, as previous studies have shown that an alkalescent penetration enhancer solution provides similar irritation to the chemical enhancers found in many ophthalmic medications [7,8]. The difference in ocular irritation suggests that mist application methodology may produce the same delivery of active ingredients as drop applications but less ocular irritation and general systemic response.

While ocular irritation leading to increased retrobulbar flow could suggest that medications with similar irritants may work more effectively with this hemodynamic change, this positive relationship between irritation and intraocular blood flow has not been demonstrated. In a similar study using both liquid drop and nebulized mist forms of diluted penetration enhancer, retinal blood flow and irritation level were evaluated. Nebulized mist produced an increase in retinal flow without ocular irritation, while the liquid drip preparation failed to produce an increase in retinal flow even while causing ocular irritation [20]. While these studies have targeted irritation alone, the effect of active ingredients on ocular hemodynamics deserves further study. While many ocular medications include irritants, the role of the active ingredients cannot be ignored. In addition to the possible intended hemodynamic changes, the active ingredient could also be an irritant, therefore bringing about hemodynamic changes similar to the ones seen in this study.

The use of nebulized mist as a targeted method of applying ocular medication may provide a non-invasive way to avoid the known limitations of liquid drop application methodologies. This advancement in drug delivery applications has the potential to increase compliance, lower costs through decreased amounts of active substance required, and potentially reduce risk of corneal damage. While the use of nebulized mist may provide a way to optimize the treatment of ocular disease, more research on new drug application methods is warranted based upon this pilot analysis.
